# Single-Cell Sequencing Analysis Based on Public Databases for Constructing a Metastasis-Related Prognostic Model for Gastric Cancer

**DOI:** 10.1155/2022/7061263

**Published:** 2022-04-27

**Authors:** Rubin Xu, Liang Chen, Wei Wei, Qikai Tang, You Yu, Yiming Hu, Sultan Kadasah, Jiaheng Xie, Hongzhu Yu

**Affiliations:** ^1^Department of General Surgery, Fuyang Hospital Affiliated to Anhui Medical University, Fuyang, Anhui, China; ^2^Department of Neurosurgery, The First Affiliated Hospital of Nanjing Medical University, Jiangsu Province Hospital, Nanjing, Jiangsu 210029, China; ^3^Second School of Clinical Medicine, Nanjing Medical University, Nanjing, Jiangsu, China; ^4^College of Pharmacy, Jiangsu Ocean University, Lianyungang, Jiangsu, China; ^5^Department of Biology, Faculty of Science, University of Bisha, Saudi Arabia; ^6^Department of Burn and Plastic Surgery, The First Affiliated Hospital of Nanjing Medical University, Jiangsu Province Hospital, Nanjing, Jiangsu, China

## Abstract

**Background:**

Although incidences of gastric cancer have decreased in recent years, the disease remains a significant danger to human health. Lack of early symptoms often leads to delayed diagnosis of gastric cancer, so that many patients miss the opportunity for surgery. Treatment for advanced gastric cancer is often limited. Immunotherapy, targeted therapy, and the mRNA vaccine have all emerged as potentially viable treatments for advanced gastric cancer. However, our understanding of the immune microenvironment of gastric cancer is far from sufficient; now is the time to explore this microenvironment.

**Methods:**

In our study, using TCGA dataset and the GEO dataset GSE62254, we performed in-depth transcriptome and single-cell sequencing analyses based on public databases. We analyzed differential gene expressions of immune cells in metastatic and nonmetastatic gastric cancer and constructed a prognostic model of gastric cancer patients based on these differential gene expressions. We also screened candidate vaccine genes for gastric cancer.

**Results:**

This prognostic model can accurately predict the prognosis of gastric cancer patients by dividing them into high-risk and low-risk groups. In addition to this, we identified a candidate vaccine gene for gastric cancer: PTPN6.

**Conclusions:**

Our study could provide new ideas for the treatment of gastric cancer.

## 1. Introduction

Although incidences of stomach cancer have declined, it is still the fifth most common tumor and has the third highest death rate [1]. The decline in cases of gastric cancer may be due to the identification of risk factors, changes in dietary habits, and improvements in the environment [2]. However, it is worth mentioning that in developing countries, gastric cancer remains a serious issue [3]. Surgical resection of tumors and lymph node dissection are the most common treatments for gastric cancer [4]. However, a large proportion of patients miss surgical treatment because the tumor is at an advanced stage or they have developed metastases [5]. Systemic chemotherapy used to be the dominant form of treatment for this group of patients [6]. However, the effects of systemic chemotherapy on advanced gastric cancer are not positive, and many patients develop drug resistance, which leads to tumor recurrence and metastasis [6]. In recent years, targeted therapy or immunotherapy seems to have emerged as the most promising treatment for advanced gastric cancer in the future [7]. HER2 inhibitors and VEGFR inhibitors of targeted therapy have achieved initially positive clinical results, and studies on immune checkpoint inhibitors are also making good progress [8]. However, our current understanding of the immune microenvironment of gastric cancer is far from sufficient. It is very worthwhile to explore the immune microenvironment of gastric cancer in order to find new biomarkers to guide the diagnosis and treatment of gastric cancer.

As a new sequencing method, single-cell sequencing analysis has attracted many researchers on account of its precision [9]. It can be said that single-cell sequencing technology is a revolutionary research method. While the bulk sequencing method ignored intercellular heterogeneity, single-cell sequencing provides a method for us to accurately analyze gene expression at the single-cell level [10]. Furthermore, we can carry out dimension reduction, clustering, and annotation of cells through single-cell sequencing. Hence, we can explore subsets of cancer cells extensively in order to understand small genomic changes. This is of great significance in the search for new biomarkers of cancer.

In this study, we divided gastric cancer cells into immune and nonimmune subtypes by single-cell sequencing analysis based on public databases. By studying the differential gene expressions of the two subtypes, we constructed the prognostic signature associated with the immune subtype of gastric cancer. This prognostic model can accurately predict the survival rate of gastric cancer patients and may provide new insights into the exploration of the immune microenvironment and immunotherapy.

## 2. Materials and Methods

### 2.1. Source of Single-Cell Sequencing Data

We downloaded the single-cell sequencing dataset of gastric cancer from the Gene Expression Omnibus (GEO) database, which contained 3 primary gastric cancer samples and paired metastatic samples.

### 2.2. Source and Processing of Transcriptome Data

Transcriptome data (STAD-Counts, STAD-FPKM), mutation data (type muTECT2), and clinical data of 407 cases of gastric cancer were downloaded from TCGA database using the “TCGAbiolinks” package. The STAD-Counts data was used for difference analysis, while the STAD-FPKM data was converted to TPM data for subsequent analysis. We obtained 348 samples with both transcriptome data and clinical data by matching the transcriptome samples with clinical data and eliminating the samples with a survival time of 0.

We also downloaded the dataset GSE62254 and the corresponding clinical data from the GEO database and finally obtained 300 samples with both transcriptome data and clinical data through matching.

### 2.3. Acquisition of Immune Cell-Related Genes (MImrGenes) Associated with Gastric Cancer Metastasis

We first calculated the percentage of mitochondrial and RBC genes in each cell using the PercentageFeatureSet function of the Seurat package. We then set screening conditions in which the number of genes per cell ranged from 200 to 7500, the percentage of mitochondrial genes was less than 10, the percentage of erythrocyte genes was less than 3, and the total expression of all genes was less than 100,000. We also excluded the effect of the cell cycle on subsequent analysis. We then normalized the data using the NormalizeData function, setting the method “LogNormalize,” scale.factor = 10000. We searched for 3000 highly variable genes and tagged the names of the top 10 genes for subsequent analysis. Subsequently, we centralized the expression of all genes through the ScaleData function for subsequent analysis. By setting the number of PCS to 7, we reduced the dimensions of the data and clustered it. We used FindMarkers to find the top 10 differentially expressed genes in each cluster and create heat maps. Next, we distinguished immune cells from nonimmune cells by marker genes of immune cells (COL2A1, PTPRC, and EPCAM) and displayed them in the form of UMAP. We then selected the immune cells and used FindMarkers to find the differential genes between the immune cells of primary gastric cancer and the immune cells of metastatic gastric cancer by setting the conditions as |deG $avg_log2FC| > 0.8 and *P* value < 0.05. Through the reduceDimension function of the Monocle package, the DDRTree method was used to reduce the dimension of the data, and the differentiation trajectory of immune cells was plotted.

### 2.4. Construction of the Prognostic Model

The expression matrix was determined by matching the MImrGenes obtained from TCGA transcriptome data. Then, through the univariate Cox regression analysis, we set the screening condition as *P* < 0.05 and preliminarily obtained genes related to prognosis. Next, these genes were obtained by the multivariate Cox regression. Finally, using the least absolute shrinkage and selection operator (Lasso) regression, we obtained the genes most associated with prognosis and built a model. The formula of the model is as follows: RiskScore = Gene1 × coef1 + Gene2 × coef2 + ⋯+genen × coefn.

We then divided the patients into high-risk and low-risk groups by calculating the median value. Then, the GSE62254 dataset was used as the validation set, and the model risk value of each sample was obtained through the predict function, which was divided into high- and low-risk groups according to the median value.

### 2.5. Evaluation of the Model

In TCGA and GEO datasets, we explored the differences in patient outcomes between the high-risk and low-risk groups. We then drew ROC curves for the model for 1, 3, and 5 years to judge the accuracy and stability of the model. In order to further explore the prognostic evaluation of gastric cancer patients by multiple models and clinical indicators, we plotted the changes in risk values, clinical data, and their combined AUC values over time using the timeROC package. To further determine whether this model is an independent prognostic factor for gastric cancer patients, univariate and multivariate Cox regressions were performed with two datasets. At the same time, in order to evaluate whether the model can distinguish gastric cancer patients effectively, we reduced the dimension of patients using the Rt-SNE package and showed the results on a t-SNE plot. We also analyzed the gene expression in the model for the high- and low-risk groups, as well as determined the proportion of gastric cancer patients who died as the risk value increased.

### 2.6. Gene Function Enrichment Analysis

We used the DESeq2 package to analyze differences between patients in the high- and low-risk groups in TCGA dataset. We set the conditions as |log2FoldChange| > 1 and *P* adjusted < 0.05 and obtained the differentially expressed genes. Then, we conducted a Gene Ontology (GO) analysis and Kyoto Encyclopedia of Genes and Genomes (KEGG) analysis through the GOplot package. In the GO enrichment analysis, the results of *P* < 0.05 were retained, and the first 10 enrichment gene sets of BP, CC, and MF were displayed in a bar chart, while the first 5 were displayed in a circle chart. In the KEGG enrichment analysis, the first 20 gene sets enriched in the pathway were displayed in the form of a bar graph, and the first 5 gene sets in the form of a circle graph.

### 2.7. Differences in Immunity between High- and Low-Risk Groups

Next, we used the “Estimate” module of the Timer 2.0 database to download the immune infiltration data obtained by 7 immune infiltration algorithms of TCGA data source patients. We then retained the immune cells that differed between the two groups and displayed them in heat maps. After this, we collected 47 immune checkpoint- (ICP-) related genes and 25 immunogenic cell death (ICD) modulators by studying the published literature and investigated the expression of these genes in the high- and low-risk groups for two datasets. Differentially expressed ICP and ICD genes were retained (*P* < 0.05) and displayed in a bar chart.

### 2.8. Single Sample Gene Set Enrichment Analysis (ssGSEA)

We collected the marker genes of macrophage and dendritic immune cells through a review of the literature and obtained the enrichment fractions of each gastric cancer sample in these two cells by the ssGSEA analysis.

### 2.9. Landscape Analysis of Gene Mutation

We constructed a mutation waterfall plot of the top 30 most frequently mutated genes in the high- and low-risk groups using the oncoplot function of the maftools R package. We then explored the relationship between these genes and the prognosis of gastric cancer. Meanwhile, in order to observe the mutation of genes in the model, we also drew a mutation map of the model genes in the high- and low-risk groups.

### 2.10. Exploration of Candidate mRNA Vaccine for Gastric Cancer

The cBioPortal site is a comprehensive database of multiple tumor datasets. In this study, the mutant genes and copy number variation genes of TCGA gastric cancer patients were obtained and visualized. The GEPIA website is an important database for cancer research. In this study, we used the GEPIA database, set conditions as |log2FC > 1| and *P* value < 0.01 to obtain the differences in gene expression of TCGA data sources of gastric cancer and normal tissues, and simultaneously analyzed the prognosis of genes in gastric cancer. The TIMER database was used in this study to explore the relationship between genes and immune cells in gastric cancer.

### 2.11. Drug Sensitivity Analysis

We calculated the 50% inhibitory concentration (IC50) of the drug in gastric cancer samples using the pRRophetic R package and gastric cancer expression matrix. Then, we obtained the drugs with different IC50 values in the high- and low-risk groups of gastric cancer by difference analysis (*P* < 0.05).

### 2.12. Construction of the Nomogram and Calibration Curve

A nomogram of the patient “TCGA-Hu-A4GY” was plotted using the regplot package to integrate risk groups with clinical features. To evaluate the accuracy of the model, we also plotted the 1-, 3-, and 5-year calibration curves of the nomogram.

### 2.13. Statistical Analysis

The rank-sum test was used for comparison between groups, with *P* < 0.05 indicating statistical significance. The R software version 4.0.3 was used.

## 3. Results

### 3.1. Single-Cell Sequencing Data Quality Control

**Figure 1:**
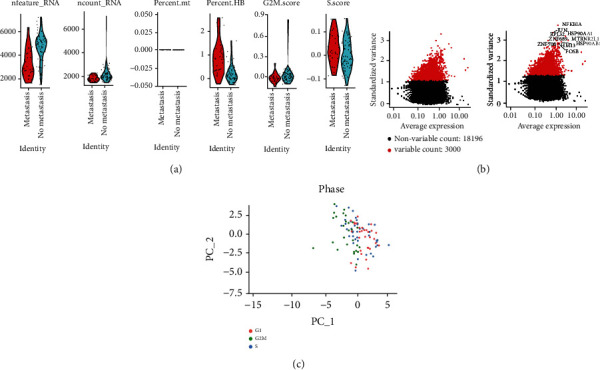
Single-cell sequencing data quality control. (a) The number of genes, percentage of mitochondrial genes, percentage of erythrocyte genes, and enrichment fraction of G2M and S phase of the cell cycle in metastatic and nonmetastatic gastric cancer cells. (b) Screening of 3000 highly variable genes. (c) Dimensionally reduced distribution of cell cycle conditions in cells.

After performing the quality control of single-cell sequencing data for gastric cancer (as shown in Figure 1(a)), it was observed that there was little difference between metastatic and nonmetastatic cells in terms of the number of cells, which ranged from 200 to 7500. The total gene expression was also concentrated, the percentage of mitochondrial genes was almost 0, the percentage of erythrocyte genes was less than 10, and the cell cycle score was similar. When combining these results with those in Figure 1(c), we found that cells in the G1, G2M, and S phases were close to each other, suggesting that cell cycle had no significant effect on subsequent analysis. We then selected 3000 highly variable genes out of 21,196 (Figure 1(b)). The red ones are highly variable genes, and the first 10 highly variable genes are marked, these being NFKBIA, JUN, HSP90AA1, RPL32, ZNF689, MTRNR2L1, ZNF700, HM13, HSP90AB1, and FOSB. We can see that the highly variable genes and the nonhighly variable genes are clearly separated.

### 3.2. Acquisition of Genes Associated with Gastric Cancer Metastasis and Immunity

**Figure 2:**
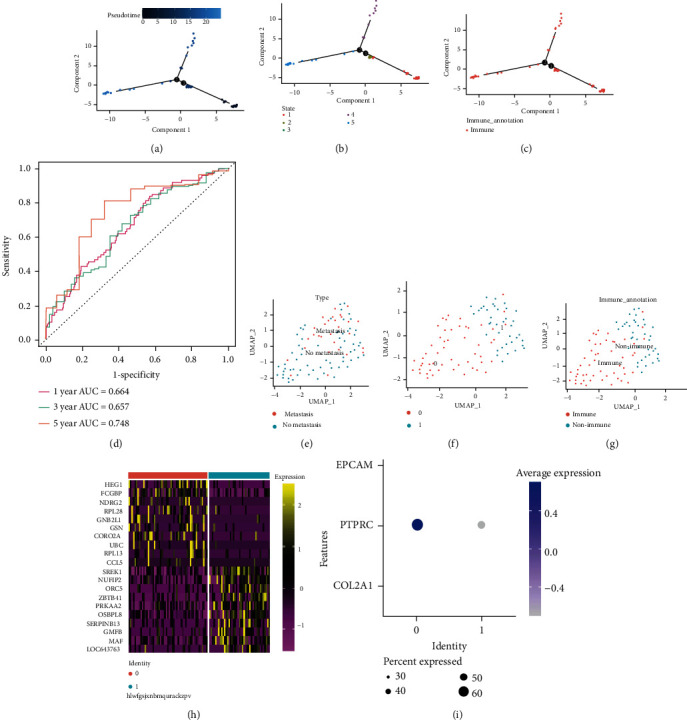
Dimensionality reduction clustering and pseudotime series analysis. (a) Spatial distribution of metastatic and nonmetastatic gastric cancer cells. It can be seen that gastric cancer cells are well divided into metastatic and nonmetastatic clusters. (b) Expression of immune cell markers in 2 clusters. (c) Spatial distribution of cluster1 and cluster0. (d) Spatial distribution of immune cells and nonimmune cells. (e) Heat map of the distribution of the top 10 differential genes in the two clusters. (f) Pseudotime series analysis. Gastric cancer-immune cells differentiate from the deeper blue to the lighter blue. (g) There are 5 states of gastric cancer-immune cell differentiation. (h) The cells analyzed are all gastric cancer-immune cells. (i) The differentiation track of metastatic gastric cancer-immune cells and nonmetastatic gastric cancer-immune cells.

After dimensionality reduction and clustering of single-cell data of gastric cancer (as shown in Figure 2(a)), we found that gastric cancer cells could be divided into two clusters: 0 and 1. We explored the expression of immune cell markers in cluster0 and cluster1 (Figure 2(b)) and, after reclustering the cells (Figure 2(c)), found that cluster0 consisted mostly of immune cells and cluster1 nonimmune cells (Figure 2(d)). We further divided all cells into metastatic and nonmetastatic cells (Figure 2(a)) and found that cluster1—that is, nonimmune cells—accounted for a large proportion of metastatic gastric cancer cells, which may indicate that an abundance of immune cells is related to the inhibition of tumor metastasis. By creating a heat map of the top 10 differentially expressed genes of cluster0 and cluster1, we found that genes HEG1, FCGBP, NDRG2, RPL28, GNB2L1, GSN, CORO2A, UBC, RPL13, and COL5 were mainly expressed in cluster0 (Figure 2(e)), while SREK1, NUFIP2, ORC5, ZBTB41, PRKAA2, OSBPL8, SERPINB13, MAF, and LOC643763 were mainly expressed in cluster1 (Figure 2(e)).

### 3.3. Pseudotime Analysis

In order to further explore the differences in immune cell differentiation between metastatic and nonmetastatic gastric cancer, we performed a pseudotime analysis. As shown in Figure 2(f), we found that the cells differentiated over time from the darker blue to the lighter blue ground.  Figure 2(g) shows that immune cell differentiation of gastric cancer has five differentiation states, with state1—in red—being the earliest differentiation state. Figures 2(h) and 2(i) indicate that in the process of immune cell differentiation of gastric cancer, the immune cells of both metastatic and nonmetastatic gastric cancer were differentiated first, the main ones being the immune cells of metastatic gastric cancer. The subsequent differentiation mainly occurred with nonimmune cells. Therefore, there are differences between metastatic and nonmetastatic immune cells in gastric cancer, which warrants further investigation. We further obtained through differential analysis 221 differential genes from immune cells of metastatic and nonmetastatic gastric cancer for subsequent analysis. These 221 MImCGenes were matched with TCGA and GSE62254 datasets, and a total of 189 overlapping genes were found for subsequent analysis.

### 3.4. Construction of the Prognostic Model

**Figure 3:**
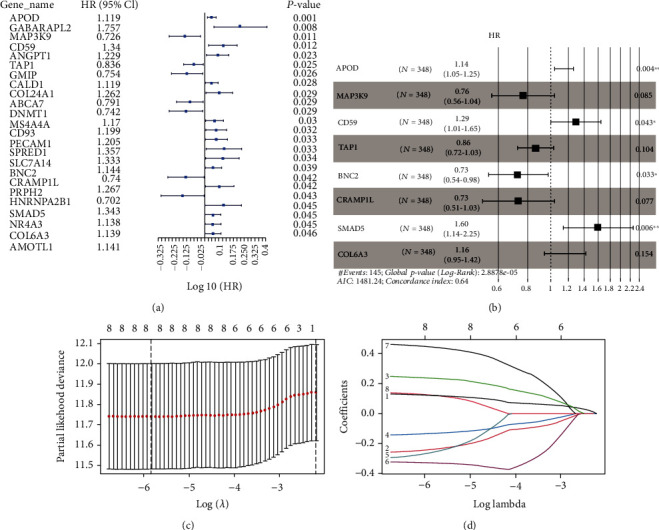
Construction of the prognostic model. (a) Univariate Cox analysis. (b). Multivariate Cox analysis. (c, d) The Lasso regression and tenfold cross-validation. Finally, a risk scoring formula composed of 8 genes was obtained. The risk value = APOD × 0.125 + MAP3K9 × (−0.245) + CD59 × 0.240 + TAP1 × (−0.135) + BNC2 × (−0.268) + CRAMP1L × (−0.327) + SMAD5 + COL6A3 × 0.123 × 0.449.

Firstly, 24 genes related to prognosis were obtained from TCGA dataset by the univariate Cox regression analysis (Figure 3(a)). The HR values of APOD, GABARAPL2, CD59, ANGPT1, TAP1, CALD1, COL24A1, MS4A4A, CD93, PECAM1, SPRED1, SLC77A14, BNC2, PRPH2, SMAD5, NR4A3, COL6A3, and AMOTL1 were more than 1, denoting poor prognosis of gastric cancer. However, the HR values of MAP3K9, TAP1, GMIP, ABCA7, DNMT1, CRAMP1L, and HNRNPA2B1 were less than 1, which indicated a better prognosis for patients. Next, we conducted multivariate Cox regression (Figure 3(b)) and screened 8 genes, these being APOD, MAP3K9, CD59, TAP1, BNC2, CRAMP1L, SMAD5, and COL6A3. Finally, the Lasso regression analysis was performed (Figures 3(c) and 3(d)). The minimum lambda value was 0.0024, and 8 genes were used to construct the model. RiskScore per patient = APOD × 0.125 + MAP3K9 × (−0.245) + CD59 × 0.240 + TAP1 × (−0.135) + BNC2 × (−o.268) + CRAMP1L × (−0.327) + SMAD5 + COL6A3 × 0.123 × 0.449. Patients were then divided into high- and low-risk groups based on median risk values for follow-up analysis. The survival analysis of 8 genes in the model was presented in Supplemental Figure S1.

### 3.5. Evaluation of the Model

**Figure 4:**
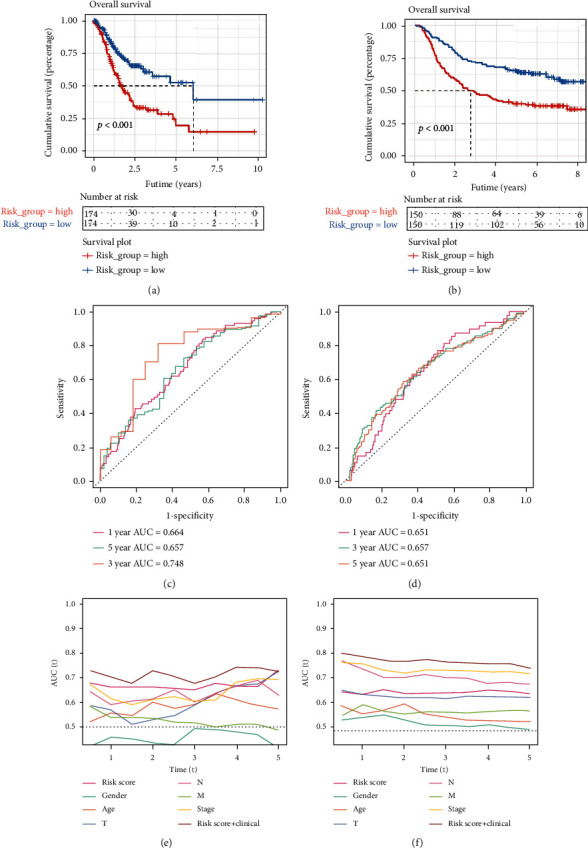
Evaluation of the model. (a, c, e) In TCGA dataset, the relationship between the model and the prognostic overall survival, the accuracy and stability of the model in predicting the prognosis of gastric cancer patients, and the comparison of the risk value and clinical characteristics of the model and the combination of the two indicators for prognosis evaluation. Similarly, (b, d, f) are the results in the GSE62254 dataset.

We evaluated the value and stability of the model in both TCGA dataset and the GSE62254 dataset. As shown in Figures 4(a) and 4(b), it was found that there was a significant difference in the prognosis between the high-risk and low-risk groups in the two different datasets (*P* < 0.01), with patients in the high-risk group showing a poor prognosis. We also found that the ROC curves for 1, 3, and 5 years were all greater than 0.65 in the two datasets, and the area under the ROC curve for 5 years in TCGA dataset was 0.748, suggesting that the model has a high degree of accuracy and is relatively stable in evaluating the prognostic outcomes of gastric cancer patients (Figures 4(c) and 4(d)). We then analyzed the value of risk and clinical data as well as the AUC value of the area under the curve of the combination of the two new indicators over time. For TCGA dataset (Figure 4(e)), it was found that the model performed well in both short-term and long-term prognoses of gastric cancer. For the GSE62254 dataset (Figure 4(f)), both the short-term prognosis assessment and the long-term tumor stage assessment performed well. For both datasets, the combined “riskScore+ Clinical” was superior to other indicators.

### 3.6. Risk Curve and t-SNE Dimension Reduction Analysis

**Figure 5:**
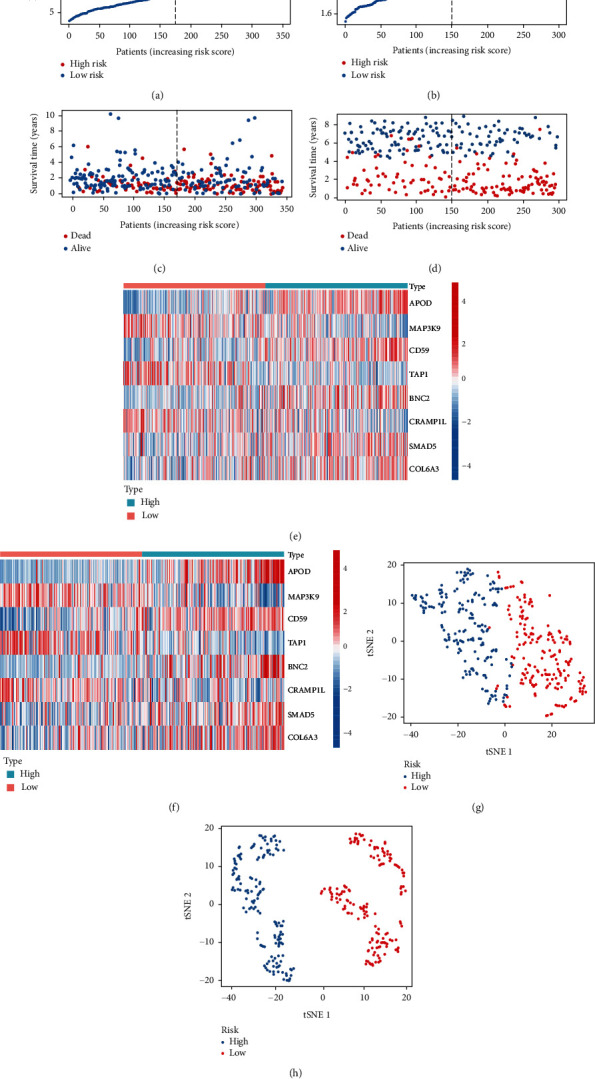
Risk curve and t-SNE dimension reduction analysis. (a, c, e) In TCGA dataset, we explored the distribution of model genes in the high- and low-risk groups and the survival of gastric cancer patients with increasing risk values. Similarly, (b, d, f) are the above results in the GSE62254 dataset. (g, h) The t-SNE dimension reduction analysis in TCGA and GSE62254 datasets, respectively.

In order to observe the gene expression in the model and the proportion of patients who died as the risk value changed, we plotted risk curves for the two datasets. As shown in Figures 5(a)–5(f), MAP3K9, TAP1, and CRAMP1L were mainly expressed in the low-risk group, while APOD, CD59, BNC2, SMAD5, and COL6A3 were mainly expressed in the high-risk group. As the risk value increased, the proportion of patients dying also increased. In order to observe the ability of the model to distinguish between gastric cancer patients, the t-SNE analysis was performed on all patients, as shown in Figures 5(g) and 5(h), and it was found that the model was able to distinguish between patients effectively.

### 3.7. Evaluation of the Independent Prognostic Value of the Model

**Figure 6:**
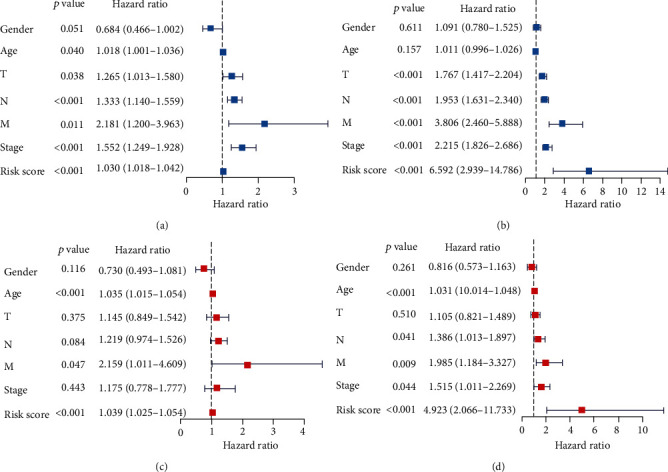
Independent prognostic value analysis. (a, c) Univariate and multivariate Cox analyses in TCGA dataset. Risk score is an independent prognostic factor for gastric cancer patients (*P* < 0.01). (b, d) Univariate and multivariate COX analyses in the GSE62254 dataset. Risk score is an independent prognostic factor for gastric cancer patients (*P* < 0.01).

We then explored the independent prognostic value of the model. Univariate (Figures 6(a) and 6(b)) and multivariate (Figures 6(c) and 6(d)) Cox regression analyses were performed on the risk score of the model and clinical indicators of patients in TCGA and GSE62254 datasets. We found that the risk scores of the models for both datasets were independent prognostic factors (*P* < 0.05).

### 3.8. Gene Enrichment Analysis

**Figure 7:**
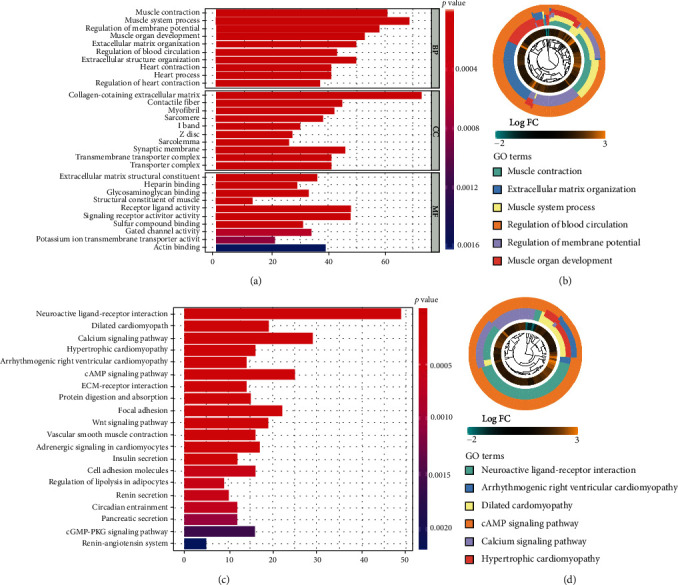
Gene enrichment analysis. (a, b) The GO functional enrichment analysis. Differentially expressed genes were mainly related to transmembrane transport across the cell membrane and receptor exchange. (c, d) The KEGG pathway enrichment analysis. They were mainly enriched in the calcium signaling, cAMP signaling, ECM-receptor interaction, focal adhesion, and Wnt signaling pathways.

In order to further explore the differences in gene function enrichment and pathway between patients in the high- and low-risk groups in the model, the GO and KEGG enrichment analyses were performed for the differentially expressed genes between the two groups. GO enrichment analysis found that differentially expressed genes were mainly related to transmembrane transport across the cell membrane and receptor exchange (Figures 7(a) and 7(b)). The KEGG enrichment analysis showed that they were mainly enriched in the calcium signaling, cAMP signaling, ECM-receptor interaction, focal adhesion, and Wnt signaling pathways (Figures 7(c) and 7(d)).

### 3.9. Immunological Differences between High- and Low-Risk Groups

**Figure 8:**
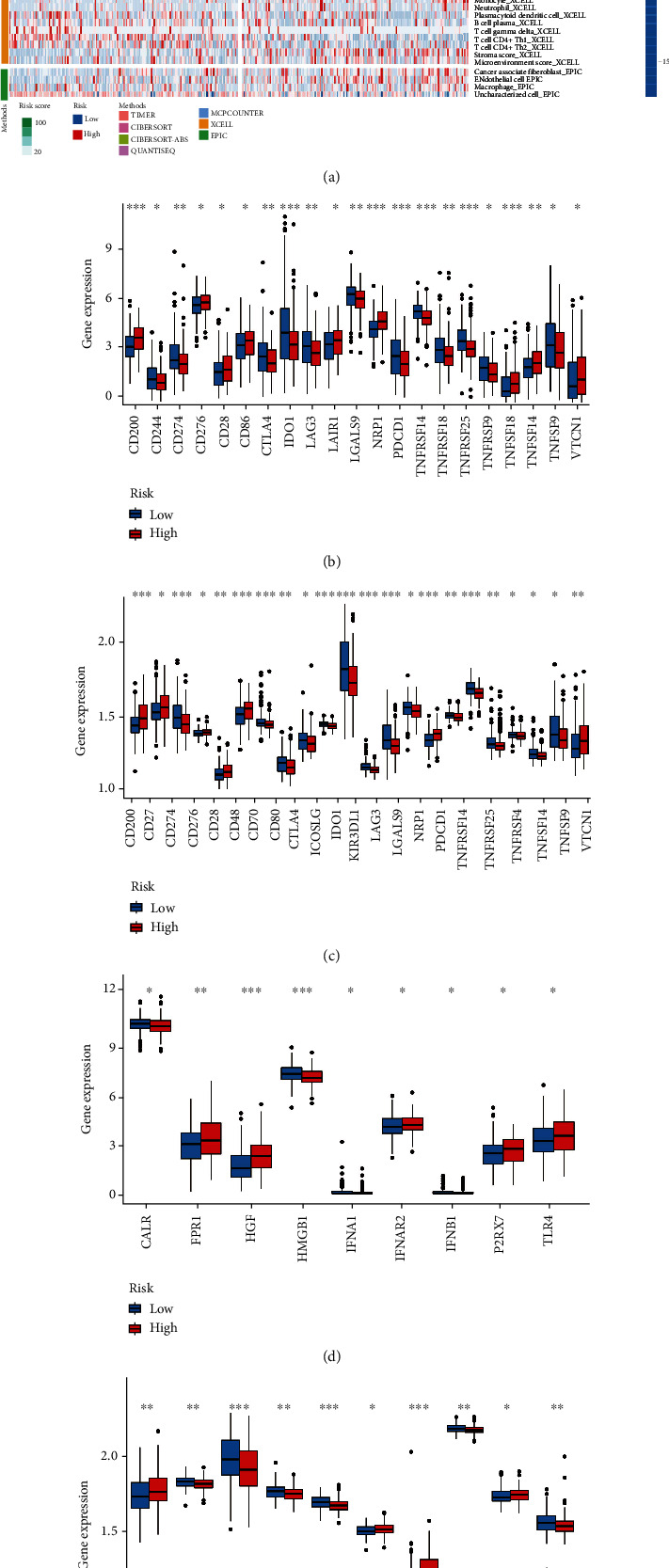
Exploration of immune microenvironment. (a) Distribution of immune cells with significantly different infiltration levels in the high- and low-risk groups in TCGA dataset (*P* < 0.05). (b, c) The expression of immune checkpoint genes in TCGA and GSE62254 datasets, respectively. (d, e) The expression of immunogenic cell death modulators (ICDs) genes in two datasets (^∗^*P* < 0.05, ^∗∗^*P* < 0.01, ^∗∗∗^*P* < 0.001).

We then explored the infiltration of immune cells in the high- and low-risk groups, selecting those with different infiltration levels of immune cells between the two groups (*P* < 0.05) and displaying the information on a heat map (Figure 8(a)). A difference was observed in the infiltration levels of immune cells between the high- and low-risk groups. The CIBERSORT method revealed that CD4 and CD8 T cells were mainly infiltrated in the low-risk group, while M2 macrophages were mainly infiltrated in the high-risk group. T cells play a central role in the human body's immune system, and the low level of T cells in the high-risk group may indicate that the poor prognosis of high-risk patients is related to the low level of T cell expression. Previous studies have reported that immune checkpoint (ICP) genes and immunogenic cell death (ICD) modulator genes play an important role in host antitumor immunity. Next, we conducted a differential analysis of immune checkpoint genes in the two datasets (Figures 8(b) and 8(c)); we found that CD200, CD276, CD28, NRP1, TNFSF4, and VTCN1 were mainly expressed in the high-risk group in both datasets, while CD274, ZDO1, LAG3, LGALS9, PDCD1, TNFSF14, and TNFSF9 were mainly expressed in the low-risk group. Meanwhile, differences in immunogenic cell death (ICD) modulator genes were also analyzed in the high- and low-risk groups (Figures 8(d) and 8(e)), and it was found that HGF and IFNAR2 were upregulated in the high-risk group, while CALR and HMGB1 were downregulated in the high-risk group.

### 3.10. Mutation Landscape Analysis

**Figure 9:**
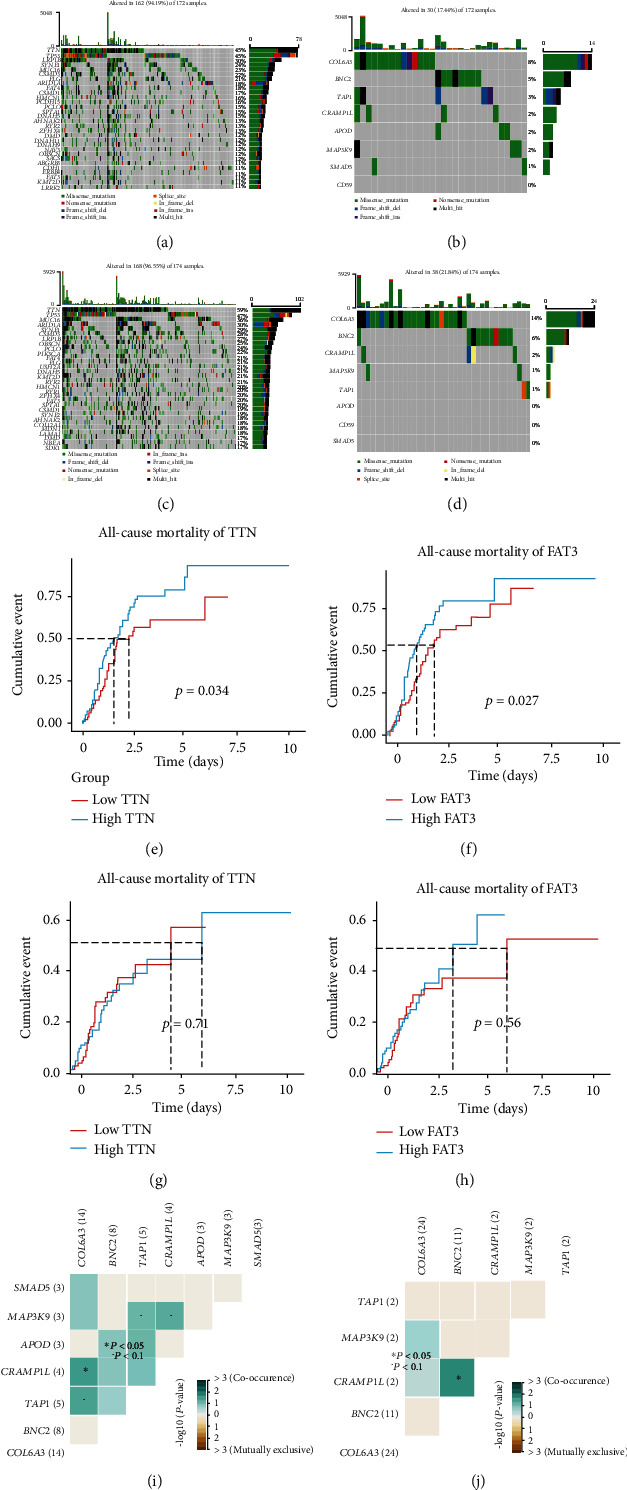
Mutation correlation analysis. (a, b) Mutation landscape of the top 30 genes in the high-risk group and mutation landscape of the 8 genes in the model. (c, d) The map of mutation landscape in the low-risk group and gene mutation in the model. (e, f) The relationship between gene TTN and overall survival in high- and low-risk groups. (g, h) The relationship between gene FAT3 and overall survival in the high- and low-risk groups. (i, j) The mutation symbiosis diagram of genes in the model in the high- and low-risk group.

Gene mutation plays an important role in the development of tumors. We used the maftools package to explore mutations in high- and low-risk groups in TCGA dataset. As shown in Figures 9(a) and 9(b), the percentage of mutations in the top 30 genes in the high-risk group was 96.55%, while in the low-risk group it was 94.19% (Figures 9(c) and 9(d)). The main mutation types were deletion mutations, and the genes showing the highest frequency of mutations were TTN and TP53. We found that the proportion of mutations in the first 30 genes was lower in the high-risk group than in the low-risk group. To further examine the causes of this, we conducted the survival analysis of the first 30 mutated genes in the high- and low-risk groups and found that the TTN and FAT3 genes correlated with patient prognosis in the high-risk group (Figures 9(e) and 9(f)), with high expression levels for both indicating poor prognosis. However, the expression of these mutated genes was not significantly associated with survival in the low-risk group (Figures 9(g) and 9(h)). These two genes may play an important role in the high-risk group of gastric cancer; they may be oncogenes of gastric cancer and could be potential therapeutic targets in the high-risk group of gastric cancer patients. Next, we analyzed the mutations of 8 genes in the model in the high- and low-risk groups (Figures 9(b) and 9(d)) and found that the percentage of mutations in the low-risk group was 21.84%, with a total of 5 genes, these being COL6A3, BNC2, CRAMP1L, MAP3K9, and TAP1 (Figure 9(d)). In the high-risk group, the mutation frequency of model genes was 17.44%, and a total of 7 genes were mutated—COL6A3, BNC2, TAP1, CRAMP1L, APOD, MAP3K9, and SMAD5, in descending order of mutation frequency (Figure 9(b)). We followed this with an exploration of the mutation symbiosis between genes in the model (Figures 9(i) and 9(j)) and found that there was mutation symbiosis between COL6A3 and CRAMP1L in the high-risk group (*P* < 0.05). COL6A3 and TAP1, TAP1 and MAP3K9, TAP1 and APOD, and CRAMP1L and MAP3K9 showed more obvious mutation symbiosis (*P* < 0.01). BNC2 and CRAMP1L displayed mutation symbiosis in the low-risk group (*P* < 0.05). Therefore, different mutational landscapes exist in the high- and low-risk groups, which may be associated with different outcomes in the high- and low-risk groups. These genes may be potential targets for immunotherapy.

### 3.11. Exploration of Candidate mRNA Vaccine for Gastric Cancer

**Figure 10:**
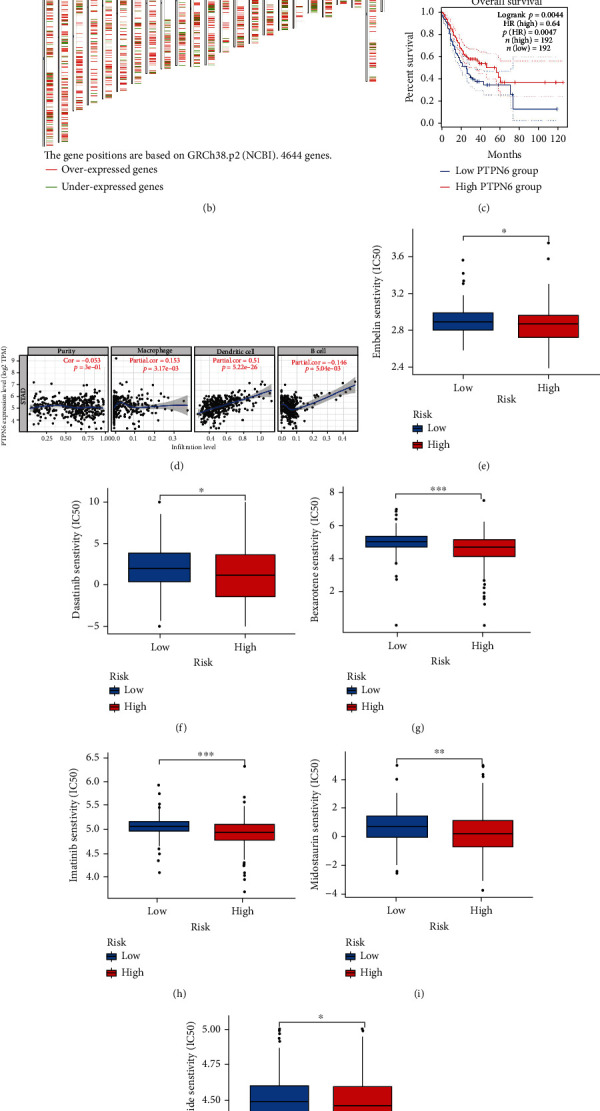
Candidate vaccine genes and therapeutic drugs for gastric cancer. (a) Distribution of genes with copy number variation in gastric cancer. (b) The distribution of different genes on chromosomes between gastric cancer and normal tissues, where red is the upregulated gene in gastric cancer and green is the downregulated gene. (c) The survival curve of PTPN6 in gastric cancer. (d) The correlation analysis between PTPN6 and tumor purity, antigen-presenting cells, and B cells. (e–j) The IC50 value of different chemotherapy drugs in the high- and low-risk groups in TCGA dataset (^∗^*P* < 0.05, ^∗∗^*P* < 0.01, ^∗∗∗^*P* < 0.001).

At present, the treatment of gastric cancer is mostly limited to surgical resection, but many people, when diagnosed, have already missed the best opportunity for treatment. The mRNA vaccine has been reported to play an important role in cancer therapy; it is therefore necessary to search for mRNA vaccine candidate genes for gastric cancer. Firstly, 16,743 mutated genes and 20,608 copy number-amplified genes of gastric cancer were analyzed on the cBioPortal website.  Figure 10(a) shows the occurrence of copy number variation in gastric cancer patients, with amplification occurring mainly on chromosomes 7, 8, and 20. At the same time, we explored the distribution of different genes in chromosomes between gastric cancer and the normal group, among which 3746 genes (marked in red) were upregulated in the gastric cancer group (Figure 10(b)). Finally, we found that both mutations and copy number variations occurred and were highly expressed in gastric cancer, and 10 genes with high expression were associated with a better prognosis. However, macrophage and DC cells were positively correlated with only one, namely PTPN6. As shown in Figure 10(c), the highly expressed gene PTPN6 suggests a more positive prognosis for gastric cancer: macrophages (COR = 0.153, *P* < 0.01, Figure 10(d)) and dendritic cells (COR = 0.51, *P* < 0.001, Figure 10(d)), as well as B cells (COR = −0.146, *P* < 0.01, Figure 10(d)). Therefore, it is likely that PTPN6 is an mRNA vaccine gene for gastric cancer, which is of great significance to the development of gastric cancer treatment. Patients in the high-risk group of gastric cancer have a poor prognosis, so we obtained 6 chemotherapy drugs through screening via the pRRophetic package, as shown in Figures 10(e)–10(j). They have different IC50 values in the high- and low-risk groups, with the IC50 values in the high-risk group being lower, so these may be candidates for the treatment of gastric cancer.

### 3.12. The Construction of a Nomogram

**Figure 11:**
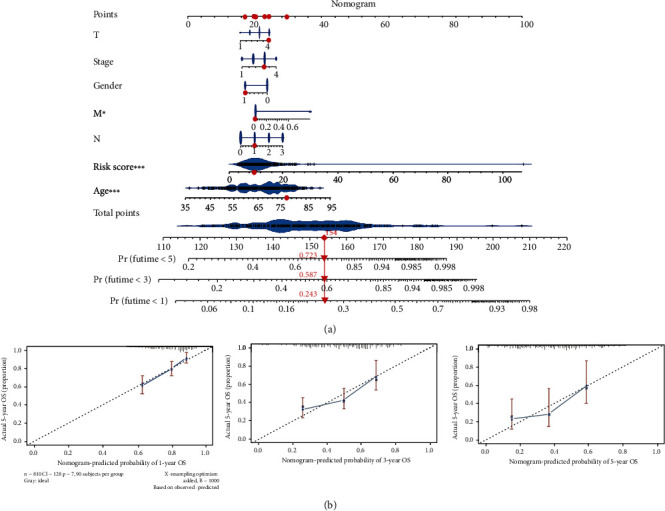
The construction of a Nomogram. (a) We constructed the Nomogram based on the risk value and clinical characteristics of the gastric cancer patient model, and predicted the 1, 3 and 5 year mortality of the patient TCGA-HU-A4Gy. (b) calibration curves of nomogram for prediction of prognosis at 1, 3 and 5 years in patients with gastric cancer

To further evaluate the prospects for survival of gastric cancer patients, we drew a nomogram combining the risk value and clinical characteristics of the model. As shown in Figure 11(a), We found that the 1, 3, and 5 mortality rates of patients TCGA-HU-a4Gy-norm were 0.243, 0.587, and 0.723, respectively. In order to verify the accuracy of survival prediction, calibration curves were also drawn (Figures 11(b)) We found that the calibration curves of 1, 3, and 5 years were all accurate, suggesting that the nomogram was an effective tool for predicting the prognosis of patients.

## 4. Discussion

As immunotherapy becomes an increasingly hot topic of scientific research, there is likewise a growing interest in tumor immunology as a focus of research [11]. Conventional treatment of gastric cancer thus far has generally been unsatisfactory [12]. Immunotherapy has achieved positive initial results in the clinical treatment of gastric cancer and has a positive outlook as a treatment method [13]. Neoantigen production, genomic instability, and viral or bacterial infection may be favorable factors for immunotherapy [14]. These factors are common in gastric cancer, so gastric cancer may prove to be an effective candidate tumor for immunotherapy [6]. It is well known that immune cell reprogramming is one of the hallmarks of tumors; consequently, the immune microenvironment of tumors is complex and changeable [15]. At present, our understanding of the tumor immune microenvironment is insufficient. It is crucial to explore the tumor microenvironment of gastric cancer in greater detail and to develop more personalized forms of immunotherapy. In addition, the in-depth study of tumor immune microenvironments and the identification of candidate vaccine genes will contribute to the development of mRNA vaccines and the development of novel tumor therapy.

In this study, we explored the differentially expressed genes of immune cells in metastatic and nonmetastatic gastric cancer by combining single-cell sequencing and transcriptome data. We subsequently constructed the prognostic model using these differentially expressed genes. This prognostic model can accurately predict the prognosis of gastric cancer patients by dividing them into high-risk and low-risk groups. In addition to this, we identified a candidate vaccine gene for gastric cancer: PTPN6. This has implications for the development of novel treatments for gastric cancer.

The prognosis of gastric cancer is usually poor, especially at an advanced stage [16]. Once gastric cancer has metastasized, treatment options are limited [17]. Therefore, it is of clinical significance to precisely evaluate the prognosis of patients with gastric cancer and accurately predict the risk of gastric cancer metastasis. Our study constructed a prognostic model of gastric cancer based on single-cell sequencing analysis and predicted the likelihood of such metastasis. The application of single-cell sequencing improves the accuracy of genetic analysis. Furthermore, the constructed prognostic model can divide gastric cancer patients into high-risk and low-risk groups in order to more effectively determine the prognosis of patients, and more personalized treatment can be provided according to these groups. This makes sense in the current era of precision medicine.

With the introduction of immune checkpoint inhibitors, the treatment of cancer has undergone landmark changes; gastric cancer is one such form of cancer to be affected by these developments [18]. The first drug to be approved by the US Food and Drug Administration (FDA) for tumor immunotherapy is ipilimumab, an anticytotoxic T lymphocyte antigen-4 (CTLA-4) antibody, which is used to treat melanoma [19]. Subsequently, PD-1/PD-L1 inhibitors were discovered and applied clinically [20]. Gastric cancer is considered an effective candidate tumor type for immunotherapy due to the frequent presence of chromosomal instability, genomic instability, microsatellite instability, Epstein-Barr virus, and Helicobacter pylori infection [21]. CTLA-4 inhibitors and PD-1/PD-L1 have been used in the treatment of advanced gastric cancer, improving the prognosis of patients to a certain extent [22]. However, there are still a considerable number of gastric cancer patients who respond poorly, tumor heterogeneity being to blame for this [23]. Therefore, we conducted an in-depth exploration of the immune microenvironment of gastric cancer and examined the differences present in this microenvironment. This is useful for increasing our understanding of tumor immunity in gastric cancer.

To summarize, we constructed an immune-related prognosis model for gastric cancer—based on single-cell sequencing analysis—which can not only accurately predict the prognosis of gastric cancer patients but also enrich our understanding of the immune microenvironment of gastric cancer. However, there are limitations to our study. We lack in vivo and in vitro trials, as well as real-world clinical cohorts to verify our conclusions; we shall make efforts to address these limitations in the future.

## 5. Conclusion

We analyzed differential gene expressions of immune cells in metastatic and nonmetastatic gastric cancer and constructed a prognostic model of gastric cancer patients based on these differential gene expressions. In addition, we screened candidate vaccine genes for gastric cancer. Our study could provide new possibilities for the treatment of gastric cancer.

## Data Availability

All data generated and described in this article are available from the corresponding web servers and are freely available to any scientist wishing to use them for noncommercial purposes, without breaching participant confidentiality. Further information is available from the corresponding authors on reasonable request.

## Abbreviations

MImrGenes: Immune cell-related genes
IC50: 50% inhibitory concentration
CTLA-4: Cytotoxic T lymphocyte antigen-4
GO: Gene Ontology
KEGG: Kyoto Encyclopedia of Genes and Genomes.

## Additional Points

*Key Messages*. What is already known on this topic: gastric cancer is one of the most common tumors at present, and the treatment effect of advanced gastric cancer is still far from ideal. What this study adds: through bioinformatics-based analysis, we explored the immune microenvironment of gastric cancer and constructed a metastasis-related prognostic model. How this study might affect research, practice, or policy: our study can be used as a reference for the prognostic assessment of gastric cancer and for the study of immune microenvironments.

## Supplementary Materials

Supplementary Materials Supplemental Figure S1: survival analysis of 8 genes in the model.
