# Supplementary figures and images for: Single-Cell Sequencing Analysis Based on Public Databases for Constructing a Metastasis-Related Prognostic Model for Gastric Cancer
Source: Appl Bionics Biomech. 2022 Apr 27;2022:7061263. doi: 10.1155/2022/7061263 (PMC9068325; doi:10.1155/2022/7061263)

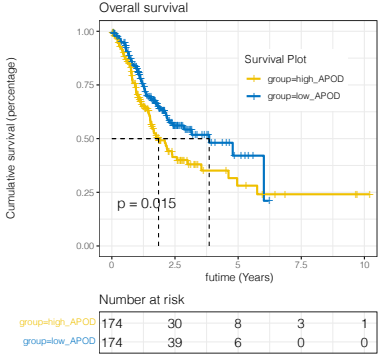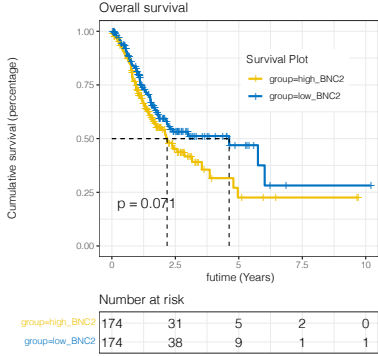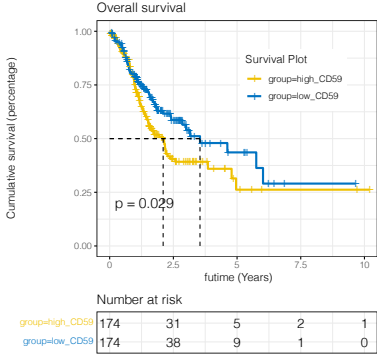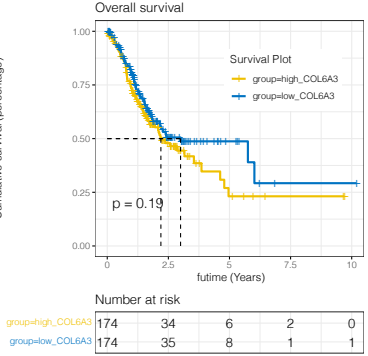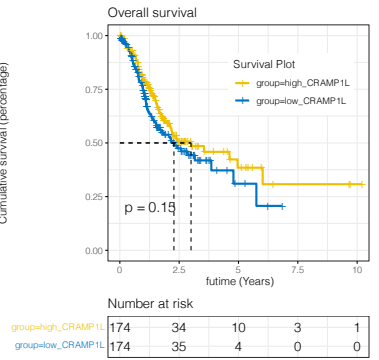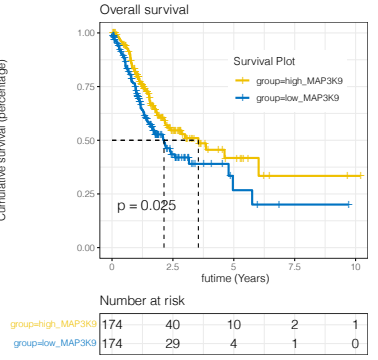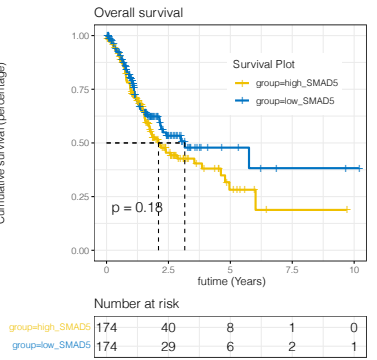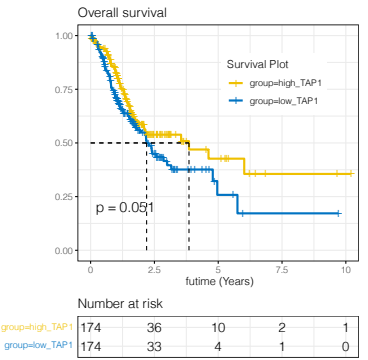

Supplement: Supplementary Materials — Supplemental Figure S1: survival analysis of 8 genes in the model. [file 7061263.f1.pdf]
